# Supplementary material for: Guiding of relativistic electron beams in dense matter by laser-driven magnetostatic fields
Source: Nat Commun. 2018 Jan 9;9:102. doi: 10.1038/s41467-017-02641-7 (PMC5760627; doi:10.1038/s41467-017-02641-7)
Supplement: Supplementary file 1 — Supplementary Information [file 41467_2017_2641_MOESM1_ESM.pdf]

Guiding of relativistic electron beams in dense matter by laser-driven magnetostatic fields – Supplementary Information

M. Bailly-Grandvaux,[1] J.J. Santos,[1,*] C. Bellei,[1] P. Forestier-Colleoni,[1] S. Fujioka,[2] L. Giuffrida,[1] J.J. Honrubia,[3] D. Batani,[1] R. Bouillaud,[1] M. Chevrot,[4] J.E. Cross,[5] R. Crowston,[6] S. Dorard,[4] J.-L. Dubois,[1] M. Ehret,[1,7] G. Gregori,[5] S. Hulin,[1] S. Kojima,[2] E. Loyez,[4] J.-R. Marquès,[4] A. Morace,[2] Ph. Nicolaï,[1] M. Roth,[7] S. Sakata,[2] G. Schaumann,[7] F. Serres,[4] J. Servel,[1] V.T. Tikhonchuk,[1] N. Woolsey,[6] and Z. Zhang[2]

[1] *Univ. Bordeaux, CNRS, CEA, CELIA (Centre Lasers Intenses et Applications), UMR 5107, F-33405 Talence, France*

[2] *Institute of Laser Engineering, Osaka University, 2-6 Yamada-oka, Suita, Osaka, 565-0871, Japan*

[3] *ETSI Aeronáutica y del Espacio, Universidad Politécnica de Madrid, Madrid, Spain*

[4] *LULI, UMR 7605, CNRS, Ecole Polytechnique, CEA, Université Paris-Saclay, UPMC: Sorbonne Universités, F-91128 Palaiseau cedex, France*

[5] *Department of Physics, University of Oxford, Parks Road, Oxford OX1 3PU, UK*

[6] *Department of Physics, Heslington, University of York, YO10 5DD, UK*

[7] *Institut für Kernphysik, Tech. Univ. Darmstadt, Schlossgartenstrasse 9, 64289 Darmstadt, Germany*

* joao.santos@u-bordeaux.fr

SUPPLEMENTARY NOTE 1:

STRONG MAGNETOSTATIC-FIELDS DRIVEN BY LASER

The first part of our experimental project was devoted to producing and characterizing strong laser-driven magnetostatic fields (B-fields) [1]. We used a long pulse (LP) laser with 1.06 µm wavelength, 500 ± 30 J energy, 1 ns flat-top duration (≈ 100 ps rise time), focused into coil-targets. These were made out of 50 µm-thick Ni-foil, laser-cut and bent to form two parallel disks, connected by a coil-shaped wire of 500 µm-diameter [see Supplementary Figure 1-a)]. The laser was incident along the surface normal of one disk, passing through the hole of the other, yielding $(1.4 \pm 0.6) \times 10^{17}\,\mathrm{Wcm}^{-2}$ of focused intensity. The target is charged by the laser-generated supra-thermal electrons escaping its potential barrier and captured on the opposite holed disk. The resulting discharge current through the wire loop closes the circuit producing a quasi-static (time-scale of a few ns), dipole-like B-field in the coil region. The laser-charging and discharge through the wire process during the laser irradiation, after this the target discharges like an RL-circuit.

Supplementary Figure 1-b) shows results for the B-field strength at the coil center, B_0, as a function of time, inferred from induction measurements at 7 cm from the coil, approximately along the coil axis, using 2.5 GHz bandwidth pick-up B-dot probes (green curves). The results are related to the discharge current intensity I and to the coil radius $a = 250$ µm, according to $B_0 \approx \mu_0 I/2a$. μ_0 is the vacuum permeability. The inferred results show reproducible charging-time, consistent with the driver LP laser duration, and peak values of $B_0 \approx 600$ T ($\pm 10\%$) and $I \approx 250$ A ($\pm 10\%$). For the earlier probing times, the B-field strength was separately measured using the Faraday rotation effect on the polarization of a probe laser beam (black square) and of the deflections of an energetic proton-beam (red circle) produced by the interaction of a short pulse (SP) laser with a Au foil placed a few mm away from the coil. These measurements confirm the inferred results using the B-dot pickup coils, at least for early times. The produced B-fields have a dipole-like spatial distribution over a 1 mm^3-scale volume. The spatial-integrated energy of the B-field at peak-time corresponds to $\approx 4.5\%$ of the driver laser energy.

Further details are described in reference [1]. The super-Alfvénic looping currents can be theoretically explained according to space charge neutralization of the driver-laser irradiated surface and the plasma magnetization [2].

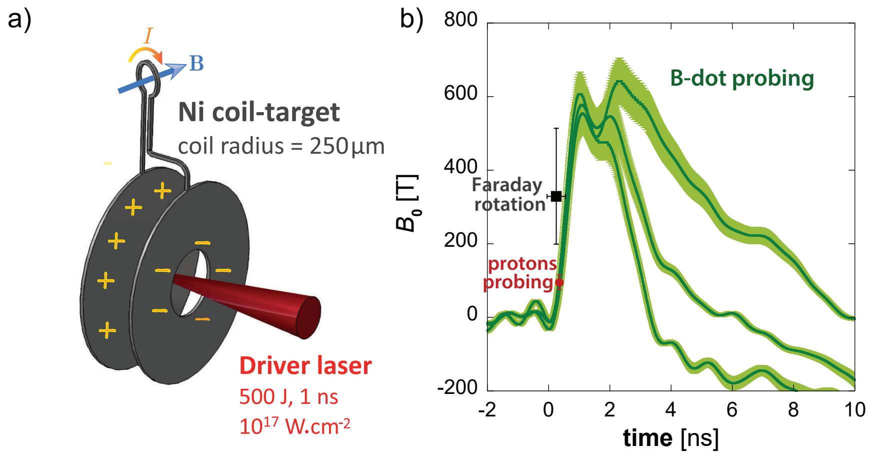

Supplementary Figure 1. **Strong magnetostatic-fields driven by laser. a)** Illustration of the B-field production mechanism with laser-driven coil-targets: An intense ns laser ejects hot electrons, corresponding to a diode-like current source. The current loops in the wire, producing a dipolar-like B-field in the coil region. **b)** Experimental results for the B-field strength at the centre of the coil, B_0, as a function of time, inferred from measurements by B-dot probes (green curves), Faraday rotation (black square) and proton-deflectometry (red circle). Details on the experimental measurements and uncertainties can be found in reference [1].

SUPPLEMENTARY NOTE 2:
MAGNETIC-FIELD DIFFUSION OVER THE TRANSPORT TARGETS

The transport-target magnetization has been predicted by simulations of the B-field resistive diffusion inside the target [3], as the B-field rises with time up to its peak-value. The model describes the penetration of external B-fields in the target material, according to the diffusion equation $\partial_t \mathbf{B} = \eta \nabla^2 B/\mu_0$, which is valid for small magnetic Reynolds number. At each time iteration, Ampère's law calculates the induced current density, from which ohmic heating is then computed for the temperature map of the target along with a new resistivity map, η. Sample results for the target position configuration i) are presented in Supplementary Figure 2-a) at $t = 0.5\,\text{ns}$ and Supplementary Figure 2-b) at $t = 1\,\text{ns}$. At $t \approx 1\,\text{ns}$ we consider the target to be fully magnetized, as the B-field spatial distribution inside the target becomes comparable to what is simulated in vacuum at the same position. Further, the B-field gradient discontinuities practically disappear at the target edges.

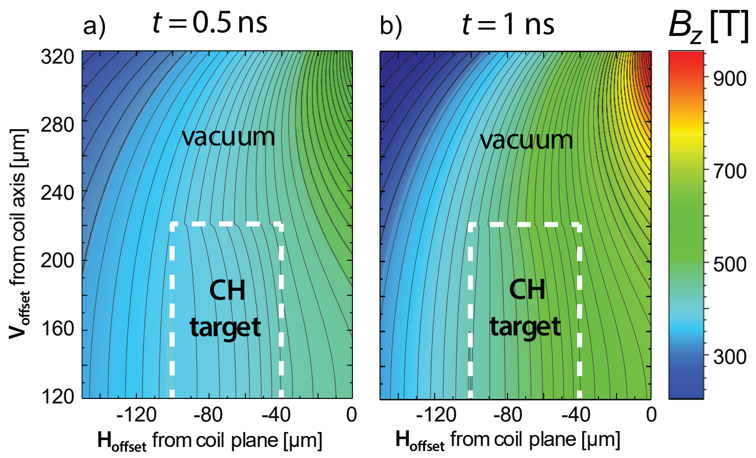

Supplementary Figure 2. B-field resistive diffusion over the transport targets. B-field spatial distribution at **a)** $t = 0.5\,\mathrm{ns}$ and at **b)** $t = 1\,\mathrm{ns}$ from axis-symmetric simulations of the B-field resistive diffusion, for the target position configuration i) with $H_{\mathrm{offset}} = -70\,\mu\mathrm{m}$ and $V_{\mathrm{offset}} = 120\,\mu\mathrm{m}$ (see setup in Fig. 1 of the main text). From the breaking on the contour lines at the target-vacuum interface, we conclude that there is a discontinuity of the B-field for $t = 0.5\,\mathrm{ns}$ and that discontinuity practically disappears at $t = 1\,\mathrm{ns}$, assumed as the time of full target magnetization.

SUPPLEMENTARY NOTE 3: PIC-HYBRID SIMULATIONS OF THE ELECTRON BEAM TRANSPORT

The hybrid method for simulating REB-transport neglects high frequency effects, hence enabling a simplification of Maxwell equations by neglecting the Poisson equation and the displacement current in the Maxwell-Ampere equation. The fast electrons composing the injected beam are modeled kinetically while the return current formed by the background electrons neutralizing the injected current, is described by Ohm's law as an inertialess fluid [4–6].

In our case, the total simulation time is set to 3.6 ps, with $t = 1.25\,\mathrm{ps}$ corresponding to the peak REB flux at the front surface. The time and spatial resolutions are 3 fs and 1 μm, respectively. Aproximately 50 millions macro-particles are used to simulate the propagation of $\sim 1 - 2 \times 10^{14}$ electrons through the target. Both collisional and ionization processes are taken into account for computing the evolution of the background electrical resistivity. For each material composing the target, the resistivity is computed as described in ref. [7] according to the Eidmann-Chimier model [8, 9].

As for the injected REB source parameters, we considered kinetic energy ε_{k} distributions between 8 keV and 10 MeV, characterized by power laws for the low energy part $\propto \varepsilon_{\mathrm{k}}^{-1.6}$ and exponential laws for the high energy part $\propto \exp(-\varepsilon_{\mathrm{k}}/T_{\mathrm{h}})$ with $T_{\mathrm{h}}^{\mathrm{i)}} = 2.0\,\mathrm{MeV}$ and $T_{\mathrm{h}}^{\mathrm{ii)}} = 1.3\,\mathrm{MeV}$, according to the conditions for each target position configuration, i) out of the coil plane, ii) at the coil plane. The characteristic hot-electron temperature is given by the ponderomotive potential [16], $T_{\mathrm{h}} = m_{\mathrm{e}}c^2(\sqrt{1+a_0^2}-1)$, where a_0 is the normalized laser intensity. The corresponding fast electron angular distribution was approximated with the following form [17]: $f_{\mathrm{h}}(\theta, r) \propto \exp\left[-(\theta-\theta_r)^2/\Delta\theta_0^2\right]$ with $\Delta\theta_0 = 55^\circ$ the dispersion angle, and $\theta_r = \arctan\left[\tan(\Theta)\, r/r_0\right]$, where $\Theta = 30^\circ$ is the mean divergence angle and the initial REB radius is $r_0 = 24\,\mu\mathrm{m}$ in configuration i) and $r_0 = 27\,\mu\mathrm{m}$ in configuration ii). These geometric and energy REB source parameters are in agreement with an experimental characterization made in the same facility with equivalent laser parameters and are supported by benchmarked simulations [7].

The results presented in the main text are calculated at the end of the simulation runs, at 3.6 ps. Yet, we observed that they differ by less than 1% from the results at 2.3 ps and 2.5 ps, respectively for the magnetized and the unmagnetized runs.

SUPPLEMENTARY NOTE 4:
CALCULATION OF CTR EMISSION (POST-PROCESSOR)

For a direct comparison between the results of the electron transport simulations and the experiment, we developed a synthetic coherent transition radiation (CTR)-emission post-processor, applied to the transport code output. The transition radiation coherence can be decomposed into temporal and spatial components [10, 13]. As the hybrid transport code continuously injects particles during the laser pulse duration, and as such does not simulate the REB longitudinal (or temporal) modulations as periodic electron micro-bunches, the transport-code - CTR post-processor suite cannot replicate the characteristic CTR harmonic spectra [14, 15]. Therefore, we assume in particular that the electron bunches produced throughout the duration of the laser-plasma interaction are identical, so that the energy spectrum and angular distribution of the REB injected in the hybrid code are representative of each individual electron bunch. The CTR spatial and angular distributions are not dependent on the number of bunches and the problem reduces then to calculating the

emission for a single electron bunch. The emission yield is calculated in arbitrary units at one wavelength, chosen consistently with the experimental observations.

For the temporal coherence, the phase difference $\phi_i - \phi_j$ of the transition radiation fields emitted at the target rear side is calculated after considering the time of flight of each individual macro-particle, i.e. we record the time difference between injection and time of arrival at the target rear side. For the spatial coherence, we assume no phase shift between macro-particles belonging to the same cell at the target rear side. This is justified by noting that the cell size in the simulation, of $1\,\mu\mathrm{m} \times 1\,\mu\mathrm{m}$, is comparable to the observed wavelength and smaller than the spatial resolution of the experimental imaging system. The coherent addition of the transition radiation fields $\mathbf{E}_i$ emitted by each macro-particle is therefore given by:

$$I_{\mathrm{TR}} \propto \sum_i |\mathbf{E}_i|^2 + \sum_i \sum_{j,j\neq i} |\mathbf{E}_i||\mathbf{E}_j| \exp\left[i(\phi_i - \phi_j)\right] \ .$$

The final synthetic image takes into account the imaging system solid angle, angle of observation and magnification factor. The finite spatial resolution is taken into account by convoluting the CTR out of the simulation with a $3\,\mu\mathrm{m}$ standard deviation Gaussian function. The results can finally be compared to the experimental CTR data. Supplementary Figure 3 shows results for the synthetic CTR for the four experimental situations analysed in the article: with and without B-field, with in config. i) the transport target outside the coil plane and in config. ii) the transport target at the coil plane. The time and space integrated CTR yields are plotted as a function of the imaging diagnostic orientation with respect to the target normal, by varying both latitude (dashed lines) and longitude (full lines). The figure also shows sample synthetic CTR images, corresponding to the lens coordinates indicated in the graphs. The four images corresponding to the experimental setup (longitude 22.5°, latitude 0°) are identified by the thicker box frames and by the dots on the graphs. The size of the dots are representative of the solid angle of the collecting lens in the experiment. The CTR yield calculation obtained either by considering the full solid angle of the lens or its center point differs by less than 1%, justifying the point-like lens approximation made for the calculations of Supplementary Figure 3.

In the case without applied B-field (blue curves and frames), the diverging REB propagation is symmetric around the injection axis. The result is a symmetric CTR emission pattern. As expected [13], peak emission in the CTR image occurs at an angle close to $1/\gamma$,

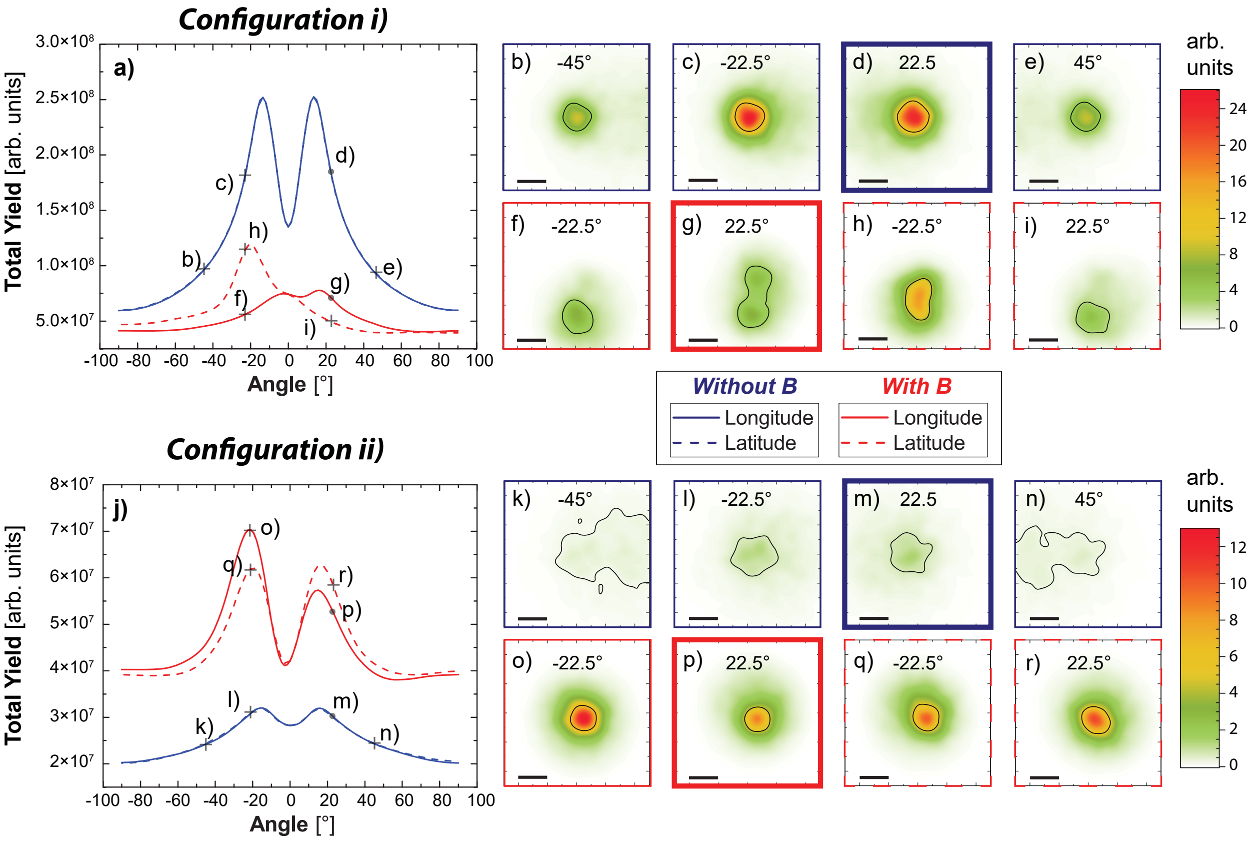

Supplementary Figure 3. **Synthetic coherent transition radiation signals for various experimental configurations.** The coherent transition radiation (CTR) yield and images are calculated from the benchmarked simulations of REB transport in the experimental conditions with (red curves and frames) and without (blue curves and frames) imposed B-field, for varying positions of the imaging lens, for **a) - i)** configuration i) with the transport target out of coil plane, and **j) - r)** configuration ii) with the transport target at the coil plane. **a)** and **j)** Total CTR yield as a function of the longitude (full lines) or the latitude (dashed lines) angles in respect to the normal of the target surface. The symbols identify the coordinates corresponding to the sample synthetic CTR images **b) - i)** and **k) - r)**, where the black bars correspond to a 20 µm scale. The four experimental configurations analysed in the main text are identified by the thicker frames and by the dot-symbols in the graphs.

where γ is the relativistic factor corresponding to the mean energy of the REB. With B-field (red curves and frames), the REB propagation is confined to a small radius and revolves at the cyclotron frequency around its symmetry axis. In configuration i), the REB axis deviates downwards due to the inclination in B-field lines, explaining the asymmetric angular

dependence of the CTR emission. The peak emission in latitude corresponds to the direction angle of the REB exiting at the target rear. In configuration ii) the angular distribution is closer to that of a beam symmetric to the target normal, yet due to the cyclotron movement, the exiting position and angle are related to the target thickness.

SUPPLEMENTARY NOTE 5:
INFLUENCE OF THE IMPOSED B-FIELD STRENGTH

As a complement of the analysis presented in Fig. 5 of the article's main text about the influence of the B-field strength, results from REB transport simulations in config. ii) with the target at the coil plane are presented in Supplementary Figure 4-a) for the CTR synthetic patterns, and in Supplementary Figure 4-b) for the time-integrated REB energy-density flux at the target's rear side, as a function of the imposed B-field strength. It is noticeable that for $B_0 \gtrsim 400\,\mathrm{T}$ both the CTR signal and rear-side REB energy-density flux are narrower and brighter in comparison to the situations with weaker or no imposed B-field, indicating a threshold on the B-field strength for efficient REB confinement. This is consistent with inducing Larmor radii for the MeV electrons already comparable to the REB source radius, $r_L \lesssim r_0 \approx 25\,\mu\mathrm{m}$. For $B_0 < 400\,\mathrm{T}$ the REB radial confinement is less efficient, yet its filamentation is already significantly smoothed compared to the unmagnetized case. The yield of the corresponding CTR patterns decreases by a factor ~ 2. Then, for the unmagnetized case, the CTR yield decreases by an extra factor ~ 2, as a consequence of the REB transport undergoing strong filamentation.

In summary, it is observed that a B-field of $B_0 \sim 100\,\mathrm{T}$ is strong enough to almost entirely smooth the REB filaments and a B-field of $B_0 \gtrsim 400\,\mathrm{T}$ is needed to radially confine the REB. Only then the produced CTR patterns at the target rear-side are comparable in size and yield (relative to the unmagnetized case) to the CTR signals measured in the experiment for the magnetized case.

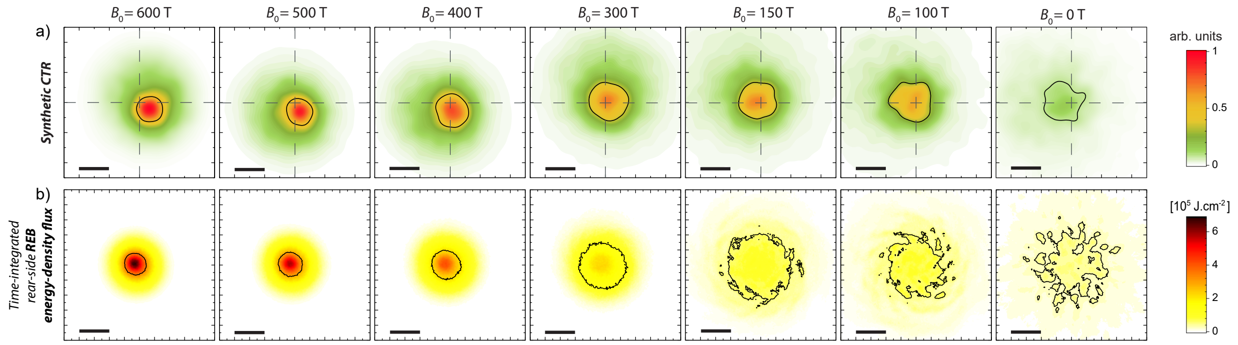

Supplementary Figure 4. Influence of the applied B-field strength on the relativistic electron beam transport. a) Synthetic CTR images (20 µm scale given by the black bars), and **b)** related time-integrated relativistic electron beam (REB) energy-density flux patterns through the target rear surface (40 µm scale given by the black bars), calculated from 3D particle-in-cell (PIC)-hybrid simulations of the REB transport in targets positioned at the coil plane. The labels B_0 stand for the different B-field amplitudes at the coil centre. The REB undergoes strong filamentation when propagating in an unmagnetized plastic target ($B_0 = 0$). For $B_0 \gtrsim 100\,\mathrm{T}$, the electrons cyclotron gyration smoothes the filaments. A significant REB radial confinement only occurs for $B_0 \gtrsim 400\,\mathrm{T}$, yielding for the average electron energy ($\approx 1\,\mathrm{MeV}$) a Larmor radius comparable or smaller than the initial REB radius of $r_0 \approx 25\,\mu\mathrm{m}$.

SUPPLEMENTARY NOTE 6:
ELECTRON REFLUXING AT TARGET'S EDGES

In this paper we were specifically interested on unfolding the B-field effects over the first REB pass, that is on the forwardly directed energy transport. REB transport simulations presented in the article's main text, matching with the experimental data, are single pass calculations, assuming non-reflective conditions for the particles reaching the target's edges. Yet, when driving thin foils with small transverse size with short, intense laser pulses, a significant part of the transported electrons can be trapped in the target volume owing to the large electrostatic fields produced by charge separation at the target's edges. The trapped electrons can reflux several times through the target, depending on their initial energy and the target's thickness and transverse size, on a time scale of several ps [18].

As an extension of the article's scope, in the perspective of, for example, driving matter to warm and dense states by the REB energy deposition [12], we present here supplementary

transport simulations considering electron refluxing and, again, compare the results in magnetized and unmagnetized conditions. To mimic the particle refluxing, we assumed specular reflection conditions at the edges of the simulation box, yet only for the fast electrons of kinetic energy below 350 keV. For the target and laser parameters of our experiment, this threshold value corresponds to a sheath potential of 350 kV calculated – according to the model proposed in ref. [19] – from the net charge of ≈ 300 nC left on target if the electrons reaching the edges were ejected into vacuum. In more details, the charge, considered as static charge at each time step, builds up from the beginning of the transport simulation and is assumed to spread over the full target's outer surface at the speed of light. A more realistic picture could have been obtained considering the dynamic evolution of the potential and trajectories of electrons escaping the target into vacuum using a full PIC description, yet this is not possible in PIC-hybrid simulations. Our approach may slightly overestimate the electron refluxing, yet the assumptions herein – as already successfully used in ref. [20] – are satisfactory to capture the effect of refluxing on the fast electrons induced heating.

Supplementary Figure 5 shows results of such PIC-hybrid simulations with electron reflective conditions at the target's edges, with the target placed at the coil plane: REB density (top row) and background electron temperature (bottom row) at the rear surface, for unmagnetized (first column) and magnetized ($B_0 = 600$ T, second column) conditions. The total simulation time was again set to 3.6 ps, and the final temperature is reached at 3 ps. As expected from the fast electrons multiple passages and extra energy deposition, the final background temperature is higher than in the results for equivalent magnetization states in absence of electron recirculation [see Fig. 3-i) and j) of the article's main text], reaching here with imposed B-field, as seen in Supplementary Figure 5-d), a peak ≈ 120 eV. This heating corresponds to impressive ~ 2 eV per joule of laser energy at the rear of the 60 μm-thick targets. The rise in background electron temperature due to the imposed magnetic confinement remains of a factor ≈ 5 as in the simulations without reflective conditions.

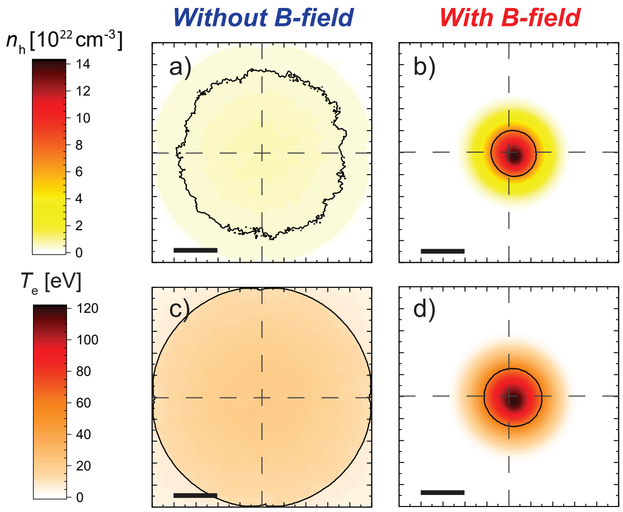

Supplementary Figure 5. Simulations of relativistic electron beam transport with refluxing conditions. 3D PIC-hybrid simulations of relativistic electron beam (REB) transport with specular reflection conditions at the target's edges. **a)**, **b)** Time-integrated rear-side REB density and **c)**, **d)** background electron temperature. The results are shown for the cases without and with imposed B-field ($B_0 = 600\,\mathrm{T}$), respectively in the first and second column. While the final temperature at the rear side rises due to the electron recirculation compared to equivalent magnetization states in absence of electron recirculation (see Fig. 3 of the article's main text), the rise in bulk electron temperature remains of a factor 5 when imposing the B-field.

SUPPLEMENTARY NOTE 7:
TRANSVERSE EMITTANCE AND PHASE-SPACE AREA OF THE ELECTRON BEAM

Emittance is usually used in conventional accelerators as a quantity describing a beam along its propagation when the motion of particles in transverse and longitudinal planes are weakly coupled. It quantifies the average spread of particle coordinates in the position-and-momentum phase-space. A statistical definition of the normalized root mean square (RMS) emittance in the (x, p_x) plane is given by :

$$\epsilon_x = \sqrt{\langle x^2 \rangle \langle {p_x}^2 \rangle - \langle x\, p_x \rangle^2}.$$

It is convenient to use momenta in dimensionless units of $\beta\gamma$ (relativistic parameters). $\langle x^2 \rangle$ defines the second central moment of the particle distribution x.

As electrons accelerated by high intensity lasers have momentum mostly directed in the longitudinal direction, we use here the emittance to describe the REB propagation over the solid-density targets in our PIC-hybrid transport simulations. For macroparticules distributions extracted from the simulations, the weight w_i of each macroparticle has to be taken into account :

$$\langle x^2 \rangle = \frac{\sum_i (w_i (x_i - \bar{x})^2)}{\sum_i w_i}$$

and

$$\langle x \, p_x \rangle = \frac{\sum_i (w_i (x_i - \bar{x})(p_{x_i} - \bar{p}_x))}{\sum_i w_i}$$

where $\bar{x}$ and $\bar{p}_x$ are respectively the weighted-average of x and p_x :

$$\bar{x} = \sum\nolimits_i (w_i x_i) \, / \sum\nolimits_i w_i \quad \text{and}$$
$$\bar{p}_x = \sum\nolimits_i (w_i p_{x_i}) \, / \sum\nolimits_i w_i .$$

The same definitions apply for the emittance calculation in the (y, p_y) plan, for the transverse vertical coordinates.

Supplementary Figure 6 plots the evolution with target depth of the REB normalized RMS-emittance in the transverse horizontal coordinates, x (solid curves), for three energy ranges within the REB energy spectrum, representative of a) the more numerous lower-energy component, b) the beam's mean energy, and c) the order of the T_{h}-parameter describing the higher-energy component. As in a conventional accelerator the magnetic guiding does not modify the beam's intrinsic divergence, the REB emittance should, in first approximation, not change under the imposed B-field as a function of the propagation depth. Yet, that is not the only mechanism determining REB transport in solid-density matter. Indeed, comparing magnetized (solid red curves) and unmagnetized transport (solid blue), the emittance does not change significantly for the two selected ranges of higher energy [Supplementary Figure 6-b) and c)], yet it is not the case for the lower energy range [Supplementary Figure 6-a)], raising considerably as a function of target depth for the unmagnetized case. This is related to the high collision diffusivity of the lower-energy electrons in the solid-density

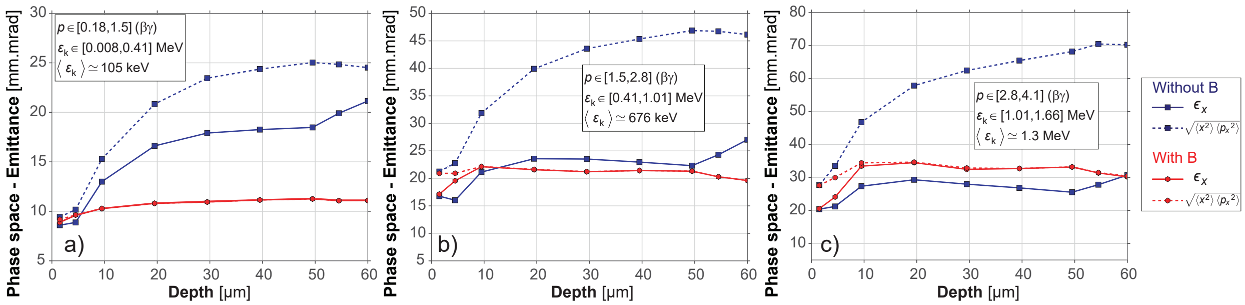

Supplementary Figure 6. Relativistic electron beam emittance and phase-space area as a function of the propagation depth. The relativistic electron beam (REB) normalized root mean square (RMS) emittance, ϵ_x (solid curves), and the phase-space area, $\sqrt{\langle x^2\rangle \langle p_x{}^2\rangle}$ (dashed curves), in horizontal transverse directions, are unfolded from the PIC-hybrid simulations with the targets at the coil plane, for cases with (red) or without (blue) imposed B-field. The two quantities are calculated for three energy ranges within the REB energy spectrum, representative of **a)** the more numerous lower-energy component, **b)** the REB population mean energy, and **c)** the T_{h}-parameter describing the higher-energy component. Each of the three chosen ranges is labeled by the corresponding momentum p and kinetic energy ε_{k} intervals, as well as by the mean kinetic energy.

material, increasing their divergence. The beam instability and disruption into filaments in the unmagnetized case can as well contribute to this. These effects are though mitigated in the magnetized case (solid red curves), where the beam emittance is fairly conserved during propagation even for the lower-energy component. The finite values mean though that if the external B-field suddenly disappears, the REB would not stay confined and would then strongly diverge.

The REB radial confinement can be appreciated for all the three energy ranges by looking instead at the evolution of the phase-space area $\sqrt{\langle x^2\rangle \langle p_x{}^2\rangle}$ [dashed curves in Supplementary Figure 6]: Without B-field (dashed blue lines) the phase-space area rises considerably with target depth due to the electrons radial spread (particularly steeply in the first $\sim 10\,\mu\mathrm{m}$) – related to the transverse momentum distribution of the REB-source, yet also to the diffusivity of the particles, mainly those of lower energy – while it is kept fairly constant in the magnetized case (dashed red curves). Interestingly, in the magnetized case, the values of the phase-space area (dashed red) and of the emittance (solid red) are approximately the

same after $\sim 10\,\mu$m depth. This happens because the imposed cyclotron effect decorrelates the electrons' position and momentum ($\langle x\, p_x \rangle \approx 0$), as seen in Fig. 4-b) of the article's main text for the beam electrons distribution at the target's rear surface, showing a fairly circular phase-space map.

---

[1] Santos, J.J. *et al.*, Laser-driven platform for generation and characterization of strong quasi-static magnetic fields. *New J. Phys.* **17**, 083051 (2015).

[2] Tikhonchuk, V.T., Bailly-Grandvaux, M., Santos, J.J., Poyé, A., Quasi-stationary magnetic fields generation with a laser-driven capacitor-coil assembly. *Phys. Rev E* **96**, 023202 (2017).

[3] Honrubia J.J. *et al.*, Enhancement of fast electron energy deposition by external magnetic fields. Journal of Physics: Conference Series **688**, 012033 (2016).

[4] Honrubia J.J. *et al.*, Laser-driven fast-electron transport in preheated foil targets. *Phys. Plasmas* **12**, 052708 (2005).

[5] Gremillet, L., Bonnaud, G., Amiranoff, F., Filamented transport of laser-generated relativistic electrons penetrating a solid target. *Phys. Plasmas* **9**, 941-948 (2002).

[6] Davies, J.R., Bell, A.R. Bell, Haines, M.G., Guérin, S.M., Short-pulse high-intensity laser-generated fast electron transport into thick solid targets. *Phys. Rev. E* **56**, 7193 (1997).

[7] Vauzour, B. *et al.*, Unraveling resistive versus collisional contributions to relativistic electron beam stopping power in cold-solid and in warm-dense plasmas. *Phys. Plasmas* **21**, 033101 (2014).

[8] Eidmann, K., Meyer-ter-Vehn, J., Schlegel, T., Huller, S., Hydrodynamic simulation of sub-picosecond laser interaction with solid-density matter. Phys. Rev. E **62**, 1202 (2000).

[9] Chimier, B., Tikhonchuk, V.T., Hallo, L., Heating model for metals irradiated by a subpicosecond laser pulse. Phys. Rev. B **75**, 195124 (2007).

[10] Santos, J.J. *et al.*, Fast-electron transport and induced heating in aluminum foils. *Phys. Plasmas* **14**, 103107 (2007).

[11] Arefiev, A., Toncian, T., Fiksel, G., Enhanced proton acceleration in an applied longitudinal magnetic field. *New J. Phys.* **18**, 105011 (2016).

[12] Perez, F. *et al.*, Enhanced isochoric heating from fast electrons produced by high-contrast, relativistic-intensity laser pulses. *Phys. Rev. Lett.* **104** 085001 (2010).

[13] Bellei, C. *et al.*, Coherent transition radiation in relativistic laser-solid interactions. *Plasma Phys. Control. Fusion* **54**, 035011 (2012).

[14] Baton, S.D. *et al.*, Evidence of Ultrashort Electron Bunches in Laser-Plasma Interactions at Relativistic Intensities. *Phys. Rev. Lett.* **10**, 105001 (2003).

[15] Popescu, H. *et al.*, Subfemtosecond, coherent, relativistic, and ballistic electron bunches generated at ω_0 and $2\omega_0$ in high intensity laser-matter interaction. *Phys. Plasmas* **12**, 063106 (2005).

[16] Wilks, S.C. *et al.*, Absorption of ultra-intense laser pulses. *Phys. Rev. Lett.* **69**, 1383 (1992).

[17] Debayle, A., Gremillet, L., Honrubia, J.J., d'Humières, E., Reduction of the fast electron angular dispersion by means of varying-resistivity structured targets. *Phys. Plasmas* **20**, 013109 (2013).

[18] Mackinnon, A.J. *et al.*, Enhancement of proton acceleration by hot-electron recirculation in thin foils irradiated by ultraintense laser pulses. *Phys. Rev. Lett.* **88**, 215006 (2002).

[19] Poyé, A. *et al.*, Physics of giant electromagnetic pulse generation in short-pulse laser experiments. *Phys. Rev. E* **91** 043106 (2015).

[20] Schönlein, A. *et al.*, Generation and characterization of warm dense matter isochorically heated by laser-induced relativistic electrons in a wire target. *Eur. Phys. Lett.* **114** 45002 (2016).
